# Acute/baseline ratios of all 3 MC mediator metabolites can enhance diagnosis and management of mast cell activation syndrome

**DOI:** 10.1016/j.jacig.2024.100399

**Published:** 2024-12-28

**Authors:** Joseph H. Butterfield, Adela Taylor

**Affiliations:** a Division of Allergic Diseases, Mayo Clinic, Rochester, Minn; b Division of Allergic Diseases, Mayo Clinic, Eau Claire, Wis

**Keywords:** Anaphylaxis, mast cell activation syndrome, histamine, N-methyl histamine, prostaglandin D_2_, (2,3-dinor)-11β-PGF_2α_, leukotriene C_4_, leukotriene E_4_

## Abstract

**Background:**

Mast cell (MC) activation syndrome (MCAS) can be a challenge to diagnose and treat despite the near continuous appearance of publications outlining specific criteria. Follow-up of the clinical responses to treatment is often lacking, and confirmation that leukotriene C_4_ (LTC_4_) is an active participant in MCAS has been overlooked.

**Objective:**

Three patients with MCAS characterized by anaphylaxis are presented to illustrate (1) the value of contemporaneous urinary mediator sampling during MCAS in addition to serum tryptase measurements and (2) substantiation of the fact that not only can LTC_4_ (measured metabolite LTE_4_) be the highest metabolite measured, but (3) blockade of the LTE_4_ receptor can contribute to symptom prevention.

**Method:**

The study methods comprised clinical review and quantitation of acute and baseline levels of tryptase and urinary MC mediators.

**Results:**

The cases of 3 patients with MCAS are reviewed. In the first case, vespid sting–induced anaphylaxis was associated with a marked increase in the LTE_4_ excretion. The addition of montelukast was instituted, and subsequent stings did not evoke symptoms. In the second case, acute measurements showed substantial increased levels of (2,3-dinor)-11β-prostaglandin F_2α_, and LTE_4_. The addition of aspirin plus montelukast prevented subsequent attacks. The third case documents a perioperative anaphylactic event with an acute/baseline LTE_4_ ratio far higher than those of tryptase or other metabolites.

**Conclusions:**

The value of measuring all 3 MC mediator metabolites during MCAS should not be overlooked. These measurements can facilitate the successful prevention of attacks. Furthermore, results from these tests show that histamine is often a minor player, whereas acute/baseline levels of the metabolites of LTC_4_ and prostaglandin D_2_ are frequently much higher, warranting nonantihistamine treatment.

## Introduction

It is recognized that the criterion standard for diagnosing mast cell (MC) activation syndrome (MCAS) in patients presenting with symptoms of MC degranulation in 2 or more organ systems is the finding of an acute elevation of serum tryptase level above baseline by 20% plus an additional 2 ng/mL. Additionally, patients with MCAS must satisfy a third criterion, namely, improvement with MC-directed treatment.^1^, ^2^, ^3^ Patients presenting with stage 1 (mild) idiopathic anaphylaxis (ie, having skin rash, itching, redness, or hives) or stage 2 (moderate) idiopathic anaphylaxis (ie, having more widespread skin rash and hives or mild angioedema of the lips or tongue) would not qualify as having idiopathic MCAS because of only single-organ involvement. Patients having the more severe idiopathic anaphylaxis, stage 3 or 4 with multiorgan involvement, must still meet the criteria of tryptase level elevation and clinical response to be diagnosed as having MCAS. Although these clinical and laboratory criteria are commonly reported, it is difficult to find reports that the third criterion for defining MCAS, namely, clinical improvement or prevention of attacks, has been satisfied following institution of treatment.^4^^,^^5^ This, in part, could be ascribed to the difficulty of prescribing appropriate therapy without knowledge that levels of MC mediator(s) are elevated during attacks. Urinary samples obtained contemporaneously with attacks can be used to measure levels of the MC mediator metabolites N-methyl histamine (N-MH), (2,3-dinor)-11β–prostaglandin F_2α_ (PGF_2α_), and leukotriene E_4_ (LTE_4_). Currently, however, despite their utility, these mediators are not currently included in the definition of MCAS. Several other mediators are synthesized and secreted by MCs; however, these are not commonly measured in clinical laboratories and are not as yet included in defining systemic mastocytosis or MCAS.^6^^,^^7^ Of the 3 urinary MC mediator measurements, those of LTE_4_ have received the least attention.^8^^,^^9^ Here we present the cases of 3 patients with MCAS, confirming the marked increases in LTE_4_ excretion and providing evidence that blockade of this product can contribute to beneficial clinical effects.

The Mayo Clinic institutional review board deemed that this report did not constitute research as defined under section 45 CFR 46.102 of the Code of Federal Regulations.

## Results and discussion

### Measurements of MC mediators

Levels of N-MH, the urinary metabolite of prostaglandin D_2_ (PGD_2_) (ie, (2,3) dinor-11b-PGF_2α_), and the stable metabolite of leukotriene C_4_ (LTC_4_) (ie, LTE_4_) were measured by liquid chromatography/tandem mass spectroscopy (normal values: N-MH, 30-200 mg/g of creatinine; (2,3) dinor-11b-PGF_2α_, <1802 pg/mg of creatinine; and LTE_4_, <104 pg/mg of creatinine [Mayo Medical Laboratories, Rochester, Minn]). Serum tryptase levels were measured by using a fluorescence enzyme immunoassay (ImmunoCAP Tryptase, Phadia AB, Uppsala, Sweden) (reference value, <11.5 ng/mL).

### Patient reports

The clinical histories and laboratory reports were obtained by chart review.

#### Patient 1

A 64-year-old man with cutaneous mastocytosis (positive for *KIT* Asp816Val), bone marrow–negative, had been followed for many years with stable disease. His history was positive for cutaneous T-cell lymphoma (stage 1A), pancreatic insufficiency (controlled with pancreatic enzyme supplement), seasonal rhinoconjunctivitis, and Barrett esophagus (treated with esomeprazole magnesium). Aside from diffuse lesions of maculopapular cutaneous mastocytosis, his physical examination findings were stable and unremarkable. While he was being maintained on a medication program of aspirin, 81 mg per day, and fexofenadine, 180 mg per day, he felt well and did not experience any recurrent or day-to-day symptoms requiring urgent care or use of his epinephrine injector.

The findings of his clinical laboratory reports had also remained stable for many years, with serum tryptase levels of 12-14 ng/mL and normal urinary levels of the MC mediator metabolites N-MH, (2,3, dinor)-11β PGF_2α_, and LTE_4_.

In 2022, however, he experienced an anaphylactic reaction following jackfruit ingestion, developing throat irritation, ocular and palmar pruritus, and subsequent loss of consciousness. Epinephrine was administered en route to the emergency department, where he was given additional epinephrine and diphenhydramine.

In 2023 following stings by 4 yellow jackets, he developed diffuse pruritus and lightheadedness. Despite using his epinephrine injector and taking oral diphenhydramine, he lost consciousness. He regained consciousness after the arrival of emergency medical services but became diaphoretic and lost consciousness again. Second doses of intramuscular epinephrine and intramuscular diphenhydramine, 50 mg, were administered, as was 450 mL of intravenous saline. The patient’s blood pressure measured 100/50 mm Hg en route to the emergency department, and his oxygen saturation was in the range of 90%. He regained consciousness by the time he arrived at the emergency department, where he was given intravenous solumedrol, 125 mg; he was observed for several hours and subsequently discharged.

His acute MC mediator levels obtained at that time are shown in Table I. The increase in the patient’s serum tryptase level qualified for the 20% + 2 metric (acute/baseline ratio is also shown), but perhaps more impressive, were the considerable increases in levels of his urinary MC mediator metabolites, especially his LTE_4_ level. When these acute values became known, the patient’s dose of fexofenadine was doubled, his dose of aspirin was increased to 81 mg twice daily, and administration of montelukast, 10 mg per day, was begun.

**Table I:** Patient 1 baseline and acute serum tryptase and urinary mast cell mediator metabolite values

Mediator (reference range)	Baseline value	Acute value	Acute/baseline
Tryptase (ng/mL), (<11.5)	12.5	72.4	5.4
N-MH (μg/g of Cr), (30-200)	120	1,205	10
(2,3-dinor)-11β-PGF_2α_ (pg/mg of Cr), (<1,802)	356	12,517	35
LTE_4_ (pg/mg of Cr), (≤104)	53	6,700	126

*Cr*, Creatinine.

Several weeks later, he sustained an additional 3 yellow jacket stings. However, in contrast to his initial reaction, this time he experienced no acute response and did not need to use his epinephrine injector.

At an allergy clinic appointment approximately 3 months later, the results of skin tests to venoms of honeybee, wasp, yellow jacket, yellow-faced hornet, and white-faced hornet were negative. The patient was maintained on his antimediator program, advised regarding vespid avoidance, and scheduled to undergo repeat skin testing at his next appointment. *TPSAB1* mutation testing was negative.

#### Patient 2

A 59-year-old female was seen for recurrent allergic reactions. Her history included anxiety, carpal tunnel syndrome, type 2 diabetes mellitus with diabetic polyneuropathy, essential hypertension, morbid obesity, and opioid use disorder. Her reactions began with palmar and plantar itch, followed by a cascade of variable symptoms, namely, hand edema, generalized urticaria, facial and tongue swelling, palpitations, a lightheaded sensation and presyncope, nausea, vomiting, and diarrhea. These symptoms resolved after about 1 hour and were followed by a day of generalized fatigue. Diphenhydramine had afforded modest relief of her acute symptoms. She reported 8 to 9 episodes during the prior year. The findings of her baseline physical examination were normal, as were her levels of serum tryptase and urinary excretion of MC mediator metabolites (Table II). Administration of cetirizine, 10 mg orally per day, was started, and she was instructed to take prednisone, 40 mg, as well as diphenhydramine and famotidine, at the next onset of symptoms.

**Table II:** Patient 2 baseline and acute serum tryptase and urinary mast cell mediator metabolite values

Mediator (reference range)	Baseline value	Acute value	Acute/baseline
Tryptase (ng/mL), (<11.5)	8.6	29.9	3.5
N-MH (μg/g of Cr), (30-200)	76	86	1.1
(2,3-dinor)-11β-PGF_2α_ pg/mg of Cr, (<1,802)	948	15,246	16.1
LTE_4_ (pg/mg of Cr), (≤104)	81	534	6.6

*Cr*, Creatinine.

One week following her initial evaluation she experienced a recurrence of acute MC activation symptoms and was seen in an emergency room setting, where her pulse rate (111 beats per minute) and blood pressure (204/103 mm Hg) were elevated. The results of her neurologic and cardiovascular evaluations were normal. A maculopapular urticarial rash with excoriations was present. The patient was observed, and contemporaneous blood and urine samples were obtained for MC mediator quantitation (Table II). These results showed substantial elevations in her acute/baseline serum tryptase values (>1.685; >20% + 2 ng/mL) and in her urinary excretion of LTE_4_ and (2,3-dinor)-11β-PGF_2α_ but only a marginal rise of N-MH level at the time of her attack. A subsequent test for the *KIT* Asp816Val mutation was negative. Prednisone was subsequently tapered and discontinued. Administration of cetirizine (mg twice daily), famotidine (20 mg twice daily), and montelukast (10 mg per day) was begun. To block prostaglandin synthesis, administration of aspirin was started at 81 mg per day and advanced to 325 mg per day. The patient declined to be tested for hereditary alpha tryptasemia but subsequently had no further attacks.

#### Patient 3

A 79-year-old male with a history of atherosclerotic heart disease, essential hypertension, treated hypothyroidism, gastroesophageal reflux disease, and prostate cancer experienced a postoperative anaphylactic reaction following cholecystectomy for recent pancreatitis and cholecystitis. Following an uncomplicated operative course, the patient developed hypotension, urticaria, pruritus, and wheezing within 5 minutes following intravenous infusion of sugammadex (200 mg). He responded to administration of supplemental oxygen, ephedrine, an intravenous fluid bolus, famotidine, hydrocortisone, and intravenous diphenhydramine. Acute samples for measurement of his serum tryptase level and a urine specimen for quantitation of MC mediator metabolites were obtained and repeated 1 month later. The results, which are shown in Table III, document acute increases in the patient’s levels of serum tryptase and all urinary metabolites, with his level of LTE_4_ higher than his level of (2,3-dinor)-11β-PGF_2α_, which in turn was higher than his level of N-MH at the time of symptoms, which is consistent with acute MCAS.

**Table III:** Patient 3 baseline and acute serum tryptase and urinary mast cell mediator metabolite values

Mediator (reference range)	Baseline value	Acute value	Acute/baseline
Tryptase (ng/mL), (<11.5)	8.4	24.1	2.9
N-MH (g/g of Cr), (30-200}	98	175	1.8
(2,3-dinor)-11β-PGF_2α_ pg/mg of Cr, (<1,802)	1006	6,108	6.1
LTE_4_ (pg/mg of Cr), (≤104)	268	10,000	37.3

*Cr*, Creatinine.

The patients reported here illustrate the importance of measuring all 3 urinary MC mediator metabolite levels to enable appropriate treatment and subsequent prevention of MCAS. There is no method to anticipate, at the time of acute presentation, which of the mediator(s) is participating in the ongoing symptoms, and a supposition cannot be predicated on the basis of the values obtained when patients are asymptomatic. Here, our 3 patients’ baseline levels of MC products were all normal.

The data presented in Tables I to III show the acute/baseline ratios for all mediators measured. The tryptase level increases exceeded the requirement of 20% + 2 for MCAS and, in addition, exceeded the more recently suggested acute/baseline tryptase value of 1.685, which has been found to improve specificity of measurements in patients with hereditary alpha tryptasemia and indolent systemic mastocytosis.^10^

In the first case, the results of mediator measurement during the patient’s sting reaction showed marked increases in his levels of all 3 metabolites and serum tryptase. The acute assay findings leading to subsequent doubling of the doses of aspirin and fexofenadine and the addition of montelukast, a leukotriene receptor antagonist, were associated with prevention of symptoms during subsequent stings.

In the case of the second patient, her tryptase increase was accompanied by increased LTE_4_ and (2,3-dinor)-11β-PGF_2α_ acute/baseline levels but only a slight increase in N-MH level. Likewise for patient 3, the level of N-MH showed a modest increase, whereas his levels of both LTE_4_ and (2,3-dinor)-11β-PGF_2α_ were again much higher. As with patient 1, the increase in level of the third patient’s LTE_4_ exceeded that of the other mediator increases.

All 3 of these patients illustrate how difficult it can be to anticipate which MC mediator(s) is being secreted when symptoms occur. Furthermore, these 3 cases demonstrate that marked elevations in the LTE_4_ level often accompany MCAS attacks, with acute/baseline ratios that can exceed those of N-MH (in patients 1, 2, and 3) and (2,3-dinor)-11β-PGF_2α_ (in patients 1 and 3). In the first 2 patients, mediator measurements suggested a preventive medication strategy that was effective. In the case of the third patient, prevention of further attacks will also depend on avoidance of sugammadex in addition to consideration of perioperative mediator blockade.

Concern continues to be raised regarding non-MC sources of LTE_4_, PGD_2_, and histamine.

Although *in vitro* studies have demonstrated that eosinophils can be a source of leukotrienes (predominantly, of leukotriene C_4_), the clinical disorders in which increases in LTE_4_ level are due in large part to eosinophils are generally characterized by a marked increase in eosinophil count and clinically differ substantially from systemic mastocytosis and MCAS.^11^^,^^12^ For example, the urinary LTE_4_ concentration during flares of eosinophilic pneumonia has been associated with the urinary eosinophil-derived neurotoxin concentration, supporting the eosinophil as a source.^13^ However, the clinical relevance of any contribution of eosinophils to measured LTE_4_ level during anaphylaxis is problematic in view of the finding that in anaphylactic patients with elevated LTE_4_ excretion, no increase in the excretion of eosinophil-derived neurotoxin has been detected.^14^

Eosinophils can also be a source of PGD_2_ when they are present in high numbers. In cases of aspirin-exacerbated respiratory disease (AERD), in which both MCs and eosinophils play prominent roles, high levels of eosinophils can be found in blood, sinus tissue, and polyps. In the case of lysyl-aspirin-stimulated eosinophils isolated from sinus tissue of patients with AERD the PGD_2_ level per 10^6^ eosinophils reached about one-third of that produced by human lung MCs stimulated by IgE.^15^^,^^16^ In the present series, the eosinophil counts were normal and there was no history of AERD in any patient.

Regarding sources of histamine, only basophils and MCs synthesize and store histamine. In the 3 patients reported here, the increase in excretion of the histamine metabolite N-MH was invariably the lowest of the mediators measured. It is possible that basophils contributed to measured levels of histamine metabolites. However, the histamine content of MCs is higher than that of basophils (as well as platelets and neutrophils), and the presence of both increased levels of tryptase (not produced by basophils) and presence of the MC- but not basophil-synthesized PGD_2_ [metabolite (2,3-dinor)-11β-PGF_2α_] support MCs as the main source.

In a recent series, reports of acute/baseline levels of urinary MC mediator metabolites that accompanied symptom-associated 20% + 2 increases in serum tryptase level were described. Unexpectedly, of the urinary MC mediator metabolites, LTE_4_ (the stable metabolite of LTC_4_) showed the greatest average increase (average acute/baseline ratio = 35.98). For all 3 metabolites, the lowest acute/baseline values that accompanied increases in tryptase level were similar (ie, approximately 1.3).^17^ A recent (2024) review by Gonzalez-Estrada et al has also shown that acute/baseline measurements of the urinary MC mediator metabolites can be useful diagnostically. In perioperative patients with anaphylaxis whose tryptase level is inconclusive, acute/baseline levels for N-MH (>1.7), (2,3-dinor)-11β-PGF_2α_ (>1.6), and LTE_4_ (>1.87) were associated with MC activation.^18^ The 3 cases reported here not only contribute evidence of the importance of measuring metabolites of histamine and PGD_2_ but also illustrate that very high urinary excretion of LTE_4_ can occur during MCAS, suggesting that a leukotriene receptor antagonist and/or a 5-lipoxygenase inhibitor should now be considered additional means to prevent MCAS attacks.

**Key messages**

• Episodes of anaphylaxis have an acute/baseline rise in the serum tryptase level greater than 1.685; however, the accompanying increases in levels of 3 urinary MC mediator metabolites are only sparsely reported in these cases.
• The acute/baseline ratios of the urinary metabolite of LTC_4_ (LTE_4_) can exceed metabolites of both histamine and PGD_2_, as well as that of serum tryptase during anaphylaxis.
• In addition to measuring urinary excretion of both histamine and PGD_2_ metabolites, documentation of acute/baseline urinary levels of LTE_4_, is warranted in patients with anaphylaxis and can contribute to therapeutic options for these patients.

## Disclosure statement

Disclosure of potential conflict of interest: The authors declare that they have no relevant conflicts of interest.
